# Liquid biopsy in pancreatic ductal adenocarcinoma from diagnostic accuracy to clinical utility

**DOI:** 10.3389/fonc.2026.1962181

**Published:** 2026-10-09

**Authors:** Konstantinos Kossenas, Nikolaos Garmpis, Nikolaos Karountzos, Christos Damaskos

**Affiliations:** 1 Laiko General Hospital, Athens, Greece; 2 Guy’s and St. Thomas’ Hospital, Division of Colorectal Surgery, London, United Kingdom

**Keywords:** artificial intelligence, circulating tumor cells, circulating tumor DNA, early detection, exosomes, extracellular vesicles, liquid biopsy, microRNA

## Abstract

Pancreatic ductal adenocarcinoma (PDAC) is often diagnosed after the opportunity for curative treatment has passed. Blood-based biomarkers offer opportunities for earlier detection and serial molecular assessment, but their value depends on the intended clinical use. This nonsystematic narrative review critically examines circulating tumor DNA (ctDNA), circulating tumor cells, extracellular vesicles, circulating RNAs, proteins and multimodal panels, including models developed using artificial intelligence. We distinguish population screening, high-risk surveillance, diagnostic evaluation, molecular profiling, treatment monitoring and postoperative recurrence assessment. High discrimination in selected case-control cohorts does not establish performance in asymptomatic populations or clinical benefit. Differences in disease stage, comparator groups, sample handling and validation design preclude reliable ranking of biomarker classes using published area-under-the-curve values. ctDNA has a more established role in selected molecular-profiling situations than in early detection; postoperative detection and longitudinal changes provide prognostic information, but do not by themselves justify treatment modification. Multimodal assays require comparison with clinical assessment and CA19–9 at prespecified thresholds, external validation, calibration and evaluation of downstream harms and costs. This review presents an intended-use framework and a summary of representative prospective studies. Limitations include purposive, non-exhaustive evidence selection and the absence of formal risk-of-bias or certainty grading. Clinical translation requires reproducible assays and evidence that biomarker-informed decisions improve outcomes. Liquid biopsy should therefore be evaluated within defined clinical pathways alongside imaging, pathology and established molecular testing.

## Introduction

Pancreatic ductal adenocarcinoma (PDAC) presents a substantial clinical burden because many patients have advanced disease at diagnosis and long-term survival remains poor. Contemporary epidemiological studies describe an increasing burden, particularly in ageing populations, although estimates vary by geographical region, calendar period and data source (1–4). Improving detection of potentially curable disease and matching treatment to tumor biology are therefore important priorities.

Diagnosis relies on clinical assessment, pancreas-protocol imaging and, when indicated, endoscopic ultrasound (EUS) with tissue sampling. Small lesions can be difficult to characterize, and biopsy specimens may be insufficient for molecular testing. CA19–9 contributes to assessment and monitoring but is unsuitable as a stand-alone screening test. Population screening of asymptomatic average-risk adults must be distinguished from surveillance of selected individuals with inherited or familial risk (5–7).

Liquid biopsy analyses circulating material that may originate from tumor cells or from the host response to cancer. It permits repeat sampling but does not guarantee complete representation of tumor heterogeneity: detection depends on shedding, anatomical disease distribution and assay sensitivity. Current approaches include ctDNA, circulating tumor cells (CTCs), extracellular vesicles (EVs), circulating RNAs and proteins (8–10). Evidence for these analytes must be considered separately for detection, molecular profiling and longitudinal monitoring.

This review examines the transition from analytical and diagnostic performance to clinical implementation. Its added value is an intended-use framework linking biomarker biology, validation design and clinical decisions, rather than a catalogue or ranking of high-performing assays. Particular emphasis is placed on multimodal integration, artificial intelligence (AI), prospective evidence and the distinction between prognostic association and demonstrated clinical utility.

## Methods

This is a nonsystematic narrative review, not a systematic review or meta-analysis. PubMed/MEDLINE, Scopus and Web of Science were searched for literature published through July 2026, using combinations of pancreatic cancer, pancreatic ductal adenocarcinoma, liquid biopsy, circulating tumor DNA, cell-free DNA, circulating tumor cells, extracellular vesicles, exosomes, microRNA, non-coding RNA, protein biomarkers, artificial intelligence, machine learning, early detection, diagnosis, prognosis and treatment monitoring. Study selection was purposive rather than exhaustive. Duplicate screening, independent study selection and formal certainty assessment were not undertaken.

The clinical synthesis focuses on human studies of blood-based biomarkers in PDAC, including diagnostic cohorts, prospective observational studies, molecular-profiling studies and published evidence syntheses. Reviews provide background; primary reports provide study-specific design and numerical results where accessible. Non-PDAC studies and conference-only reports are not used to support PDAC-specific performance claims. Translational and technical work may inform biological mechanisms or assay limitations, but analytical feasibility is not treated as clinical validation. This distinction allows emerging platforms to be discussed without conflating technical feasibility with clinical readiness.

Studies are selected purposively to illustrate different analytes, clinical applications and validation designs. **Table 1** maps biomarker classes to potential uses and interpretive limitations; **Table 2** contextualizes selected diagnostic platforms; **Table 3** includes representative prospective diagnostic and longitudinal studies plus a published study protocol. These tables are not exhaustive and selection is not based on a prespecified performance threshold. Design characteristics not explicitly reported in the cited sources were not inferred. Protocol targets are distinguished from completed results.

**Table 1:** Biomarker roles and evidence limitations.

Analyte	Biological information	Potential clinical role	Evidence context	Interpretive limitation
ctDNA	Somatic variants and epigenetic or fragment features	Selected genotyping; prognosis; monitoring and postoperative risk	Diagnostic and longitudinal cohorts (21, 25, 29)	Low shedding causes false negatives; mutation origin and assay coverage matter
CTCs	Intact cells and phenotype	Metastatic biology; exploratory prognosis and monitoring	Heterogeneous enrichment studies (15, 28)	Counts depend on capture method; predictive utility not established
EVs	Vesicular proteins and nucleic acids	Exploratory diagnosis, prognosis and monitoring	Selected diagnostic studies and pooled subgroups (30, 32)	Non-tumor EVs and co-isolates; isolation methods differ
Circulating RNAs	Tumour and host transcriptional signals	Exploratory detection and monitoring	miRNA evidence syntheses and transcriptomic models (29, 34)	Hemolysis, normalization, cell composition and model validation
Proteins and metabolites	Tumour and systemic responses	Diagnosis and clinical risk assessment	Case-control proteomics; prospective metabolomics (35, 37)	Inflammation, cholestasis and metabolic confounding
Multimodal panels	Complementary signals across analytes	Application-specific detection or stratification	Mostly development/validation; prospective protocols (27, 41)	More complexity does not establish net benefit; requires a locked assay

Evidence map, not a head-to-head comparison. Study designs, populations, thresholds and validation methods differ. Clinical roles are potential uses unless explicitly identified as established; performance in one use does not validate another.

**Table 2:** Context for selected diagnostic platforms.

Study and assay	Population and design	Validation	Reported result	Principal limitation
Malakian et al. (33) DEP DNA and Glypican-1	36 samples; benign disease/IPMN and cancer; 3 stage I–II cases	Blinded laboratory analysis; no independent external cohort reported	Sensitivity 92%; specificity 83%; AUC 0.93	Small exploratory cohort; age-subgroup AUC is not screening validation
HELP-15 (34) 15-mRNA RT-qPCR and ML	1,504 participants; 417 PDAC; 76 resectable; 1,087 non-disease controls	Internal training/tuning/test separation	AUC 0.956 overall; 0.968 resectable; 0.985 combined with CA19–9 in resectable disease	Healthy-control predominance; internal test performance is not external validation
Mellby et al. (35) 29-protein antibody microarray	Discovery: 443 PDAC, 888 controls; US validation: 143 PDAC, 219 controls	Independent case-control cohort; 90 stage I–II validation cases	Stage I–II versus controls AUC 0.96 in both cohorts	Early-stage cases already diagnosed; asymptomatic screening utility unproven
Wang et al. (36) Single-molecule LIF, CA19–9 and CA125	Technical clinical-sample demonstration; cohort details not verified here	Independent clinical validation not verified here	Clinical-performance estimate not reproduced	Cohort composition and independent validation were not reported in the abstract

Purposively selected examples, not an exhaustive review or a platform ranking. AUC denotes area under the ROC curve; DEP, dielectrophoresis; IPMN, intraductal papillary mucinous neoplasm; ML, machine learning. Unknown information is not assumed to be absent from the full publication. All values retain their original study context.

**Table 3:** Representative prospective studies and a published protocol.

Study and application	Population	Analytes and platform	Endpoint and validation	Results or remaining question
PANLIPSY (41) Diagnostic development Protocol published 2024	Planned: 215 PDAC, 25 benign pancreatic disease, 115 healthy controls	CTCs, ctDNA, EVs and other signals; multimodal laboratory assays and AI	Signature discovery; blinded validation of 30 resectable PDAC and 30 healthy controls	No completed accuracy results in cited protocol; benign controls absent from planned final validation
METAPAC (37) Lesion evaluation Published 2025	23 centers; primary analysis 1,129 participants: 489 PDAC and 640 controls	Plasma metabolites and CA19-9; targeted LC–MS/MS	Prospective masked validation of diagnostic signatures in an enriched population	i-Metabolic AUC 0.846; specificity 90.4%, sensitivity 67.5%; clinical-impact benefit not established
Groot et al. (25) Recurrence surveillance Published 2019	59 resected PDAC; 290 plasma samples	KRAS mutations; droplet digital PCR	Prospective longitudinal clinical validation; recurrence and survival associations	Median lead time 84 days; observational, not a ctDNA-directed treatment trial
Bernard et al. (26) Treatment monitoring Published 2019	194 localized/metastatic cases; 425 blood samples	ctDNA and exosome-derived DNA; KRAS droplet digital PCR	Prospective longitudinal sampling; progression and survival associations	Analyte-dependent findings; external thresholds and intervention benefit require validation

Publication-based snapshot, not a live recruitment-status inventory. Protocol sample sizes are planned; other counts are from published studies. Endpoints are the reported main objectives, not an assertion of a formally registered primary-endpoint hierarchy. LC–MS/MS, liquid chromatography–tandem mass spectrometry. Prospective design does not by itself establish clinical utility.

Evidence was appraised qualitatively according to patient selection, disease-stage distribution, comparator groups, reference standards, threshold selection, blinding, validation methods and clinical applicability. No formal QUADAS-2 assessment, duplicate data extraction, GRADE certainty assessment, pooled analysis or indirect ranking was performed. Evidence is therefore described according to study design and applicability rather than assigned a numerical quality score. Selection and publication bias remain important limitations.

## Clinical rationale and advantages of liquid biopsy

Liquid biopsy can complement imaging and pathology by enabling repeated molecular measurements with a peripheral blood draw. Its practical advantages are greatest when tissue sampling is difficult or when serial observations are needed (11–18). However, easier sampling does not ensure that the result changes care, and a technically detectable signal may not be specific to malignancy.

Three questions should be kept separate: can the assay measure its target reproducibly (analytical validity), does the result identify disease or predict an outcome in the intended population (clinical validity), and does acting on the result improve patient outcomes or reduce harm (clinical utility)? These questions require different studies. A high area under the receiver operating characteristic curve (AUC) primarily addresses discrimination and cannot answer the third question (19–21).

For selected patients with established advanced cancer, validated plasma ctDNA testing can help identify actionable alterations when tissue is unavailable or timely testing is important. This molecular-profiling use should not be conflated with diagnosing an undetected pancreatic cancer. A non-informative plasma result may require tissue testing because low tumor fraction can produce false negatives; plasma testing also does not replace indicated germline testing. ESMO recommendations distinguish this use from screening, molecular residual disease-directed treatment and early treatment-response assessment, for which evidence of clinical utility remains insufficient for routine adoption (21, 22).

## Clinical applications and intended populations

### Population screening and high-risk surveillance

Population screening tests asymptomatic people without a recognized high-risk condition. In this low-prevalence setting, even modest false-positive rates create substantial downstream investigations, and no assay discussed here establishes a population screening benefit. High-risk surveillance instead follows people with selected pathogenic germline variants or a strong family history. MRI/MRCP and EUS remain the principal surveillance modalities in expert programs; blood biomarkers are investigational additions that should be assessed for interval cancers, stage at diagnosis, false-positive procedures and outcomes (23, 24).

### Symptomatic patients and indeterminate lesions

Diagnostic evaluation concerns patients with symptoms, abnormal imaging or other evidence suggesting pancreatic disease. The clinically relevant controls include chronic or autoimmune pancreatitis, benign cysts, biliary obstruction and other malignancies. Testing an indeterminate lesion asks whether a biomarker changes the need for repeat imaging, EUS-guided sampling or surgery. The reference standard should include pathology where obtained and sufficient clinical follow-up in patients managed without surgery. A negative blood test must not delay an otherwise indicated diagnostic work-up (5, 22).

### Established disease and postoperative follow-up

After PDAC is established, molecular profiling seeks actionable alterations; monitoring asks whether treatment response or progression can be detected; postoperative testing seeks evidence of molecular residual disease; and recurrence surveillance evaluates disease return over time. These applications have different sampling schedules, thresholds and acceptable error rates. Among the uses discussed, selected ctDNA genotyping has the clearest established route into clinical care. Monitoring and postoperative risk stratification have prospective association data, whereas changing treatment solely because of these signals requires interventional evidence (21, 25, 26).

### Prevalence and predictive values

Positive predictive value (PPV) depends on prevalence as well as sensitivity and specificity: PPV = sensitivity × prevalence/[sensitivity × prevalence + (1 − specificity) × (1 − prevalence)]. As an illustrative calculation, not an estimate of PDAC prevalence, assume 90% sensitivity and 99% specificity in 100,000 people with 0.1% prevalence. The test yields 90 true positives, 999 false positives, 10 false negatives and 98,901 true negatives: PPV is 8.3% and negative predictive value (NPV) is approximately 99.99%. At an assumed 1% prevalence, PPV rises to 47.6% with the same sensitivity and specificity. A high NPV in a rare disease can therefore coexist with many false positives and missed cancers. These calculations assume fixed test characteristics; in practice, spectrum effects can change sensitivity and specificity when a healthy-control study is transported to patients with benign pancreatic disease. Predictive values calculated directly from an artificially balanced case-control sample do not represent a screening population.

## Major liquid biopsy biomarkers

The major analytes differ in biological origin, tumor specificity and practical purpose. Diagnostic biomarkers distinguish disease states; prognostic biomarkers associate with future outcomes; predictive biomarkers identify differential benefit from a particular therapy; monitoring biomarkers track change; and molecular-profiling assays identify alterations relevant to treatment or trial eligibility. A marker can serve more than one role, but evidence for one role does not validate the others. **Table 1** summarizes these distinctions (9, 13–16, 27, 28).

### Circulating tumor DNA

ctDNA is the tumor-derived fraction of circulating cell-free DNA and can contain somatic mutations, copy-number changes, methylation patterns or other tumor-associated features. Mutation-based assays, including KRAS-focused methods, are relevant to molecular profiling and longitudinal assessment, while epigenetic and fragmentomic approaches seek complementary detection signals. Total cell-free DNA concentration is not equivalent to ctDNA because non-malignant tissues also contribute DNA (14, 28).

Low shedding limits mutation detection in small tumors and after treatment. A negative result therefore cannot reliably exclude early PDAC or residual disease. The pooled sensitivity of 65% and specificity of 94% reported for ctDNA in a recent early-diagnosis meta-analysis describe heterogeneous assays and populations, rather than a universal property of ctDNA (29). False-positive variant attribution can also arise from clonal hematopoiesis; appropriate variant filtering and, where relevant, paired leukocyte analysis are needed (21).

### Circulating tumor cells

CTCs provide intact cellular material for phenotypic and molecular analysis and may inform metastatic biology. Associations with prognosis and treatment response are biologically plausible, but rare cell numbers, variable enrichment and inconsistent definitions complicate reproducibility (9, 14–16). A prognostic association with CTC burden should not be described as evidence that a specific treatment will benefit a patient.

Epithelial-marker enrichment can miss cells that have reduced epithelial-marker expression, while size-based or marker-independent approaches capture different cell populations. Counts from different platforms are therefore not interchangeable. CTC assays remain investigational for PDAC diagnosis and treatment selection; claims of added value require assessment beyond established clinical prognostic factors (15, 28).

### Extracellular vesicles and exosomes

EVs carry proteins and nucleic acids from malignant and non-malignant cells. Their protected cargo offers opportunities for biomarker development, but abundance does not establish tumor specificity. “Exosome” refers to a biogenesis-defined EV subtype and should not be used for every small-vesicle preparation when endosomal origin has not been demonstrated. Isolation and characterization should follow appropriate EV reporting principles (20, 30, 31).

The frequently cited sensitivity of 93%, specificity of 92% and summary AUC of approximately 0.98 arose from the six exosome studies within Zhu et al.’s 19-study meta-analysis, not from 19 exosome studies (32). These subgroup results do not establish superiority over ctDNA or CTCs because studies differed in patient selection, targets and controls. Conflicting findings for individual EV markers can reflect co-isolated proteins or lipoproteins, differing isolation methods and inflammation-related signals; validation against benign pancreatic disease is essential (28, 33).

### RNA and protein biomarkers

Circulating miRNA signatures may detect host and tumor-associated changes, but hemolysis, blood-cell composition, extraction and normalization can alter measured concentrations. Zheng et al.’s review included 43 studies and 19,326 participants across biomarker categories, with 2,749 stage I–II cases; those totals must not be interpreted as the miRNA subgroup sample size. Its miRNA estimates were sensitivity 88% (95% CI 79–93%), specificity 91% (82–95%) and AUC 0.95 (29). These are pooled diagnostic estimates, not performance established in prospective population screening.

Protein and metabolite panels can capture complementary tumor and host responses and may be easier to integrate into laboratory workflows than some rare-analyte assays. However, inflammation, obstructive jaundice, metabolic disease and other cancers can affect these signals. The contribution of each component must be tested against CA19–9 and clinical information in the same intended-use population (27, 28). RNA, EV and protein associations with outcome remain distinct from validated predictive biomarkers for selecting a therapy (30).

## Diagnostic performance and emerging multimarker platforms

Published AUCs cannot be used to rank platforms tested in different populations. Healthy controls make cancer discrimination easier than chronic pancreatitis or indeterminate cystic disease; advanced cancers may shed more biomarker than small resectable tumors. Reference-standard differences, selected thresholds, retrospective sampling, batch effects and derivation-set optimism can also explain apparently conflicting results. Prospective sample collection alone does not ensure an unbiased diagnostic cohort if cases and controls are selected separately. Comparisons are most informative when assays are evaluated head-to-head, blinded to the reference standard, with prespecified thresholds and independent validation.

Meta-analyses provide useful context but inherit the limitations of their component studies and may include overlapping cohorts or multiple thresholds. Pooled sensitivity and specificity, and summary ROC AUCs, are not equivalent to the performance of one deployable test. In particular, the indirect subgroup estimates in references (29, 32) should be interpreted as hypothesis-generating. **Table 1** consequently avoids a cross-platform performance league table; **Table 2** reports selected study-specific results alongside design constraints.

Malakian et al. evaluated nanoparticle-associated DNA and Glypican-1 in a blinded 36-sample cohort that included benign pancreatic disease and IPMN. The report gave sensitivity 92%, specificity 83% and AUC 0.93, increasing to 0.97 in patients older than 50 years (33). Only three stage I–II cases were listed. The small sample and subgroup analysis limit precision and generalizability.

The large single-center HELP-15 study analyzed 1,504 participants, including 417 PDAC cases, of whom 76 were resectable, and 1,087 non-disease controls. Reported test-set AUCs were 0.956 for all PDAC and 0.968 for resectable disease; the combined HELP-15/CA19–9 AUC of 0.985 applied to resectable disease (34). The predominance of non-disease controls and internal data splitting limit inference about external performance. Mellby et al.’s 29-protein signature achieved AUC 0.96 for stage I–II disease in discovery and independent validation cohorts, but both were case-control studies (35). External validation strengthens transportability evidence without establishing screening utility.

Wang et al.’s single-molecule protein assay illustrates technical progress in measuring low- and high-abundance proteins together (36). However, the abstract does not report sufficient information on cohort composition, sample size or independent validation to allow confident interpretation of clinical performance. Analytical sensitivity and proof-of-concept classification should not be equated with clinical readiness.

A more clinically relevant example is METAPAC, a prospective, masked study across 23 centers. Among 1,129 patients with pancreatic lesions in the primary analysis, the i-Metabolic signature had AUC 0.846 versus 0.799 for CA19-9; specificity increased from 79.1% to 90.4%, while sensitivity decreased from 81.8% to 67.5% (37). Thus, a higher AUC can conceal a clinically important trade-off at the operating threshold. This diagnostic study does not establish population screening benefit or improvement in survival. Its results should not themselves be used to rank metabolomics below assays tested against healthy controls.

### CA19–9 and incremental value

CA19–9 expression depends on Lewis antigen biology; some individuals produce little or none, and a normal concentration cannot exclude PDAC. Early-stage disease may also produce limited elevation. Conversely, cholestasis, cholangitis and pancreatitis can elevate CA19–9 without malignancy. Clinical interpretation should consider biliary obstruction, timing relative to drainage and disease burden (5, 22, 28).

An improved combined-panel AUC does not necessarily demonstrate useful information beyond CA19-9. Evaluation should compare a prespecified clinical model plus CA19–9 with the same model plus the new assay, assess sensitivity at a clinically acceptable fixed specificity, and report calibration and decision consequences. Performance should be examined separately in patients with normal CA19-9, obstructive jaundice and relevant benign disease. These are proposed evaluation requirements, not analyses newly performed in this review.

## Molecular profiling, prognosis and longitudinal monitoring

Molecular profiling asks whether an assay identifies a therapeutically actionable alteration, not simply whether ctDNA is detectable. The clinical significance depends on the alteration, assay validation, treatment context and evidence supporting the matched therapy. Tumor fraction, coverage and detection of fusions or copy-number changes can limit plasma testing. A positive result warrants appropriate clinical interpretation, while a negative result may be non-informative. Putative germline findings require confirmatory testing through an appropriate pathway (21).

During therapy, falling ctDNA may correlate with response and persistent or rising levels with progression, but these associations are affected by baseline detectability, treatment timing, tumor burden and assay limits. Bernard et al. prospectively collected 425 samples from 194 patients with localized or metastatic disease. Their findings were not uniform across analytes: in potentially resectable disease, changes in exosome-derived DNA associated with progression after neoadjuvant treatment, whereas ctDNA did not show the same outcome association (26). This illustrates why one circulating analyte cannot be assumed to substitute for another. Treatment-response association is not proof of differential benefit from a particular regimen.

After resection, detectable ctDNA can identify patients at increased risk of recurrence. Groot et al. studied 290 longitudinal plasma samples from 59 patients; detection during follow-up anticipated recurrence with sensitivity 90% (95% CI 74–98%), specificity 88% (62–98%) and a median lead time of 84 days (25). These estimates describe recurrence surveillance in a selected surgical cohort, not screening accuracy. The wide intervals and small cohort warrant caution.

Postoperative sampling must account for transient non-tumor DNA release, variable timing and adjuvant treatment. Tumor-informed assays may improve specificity but require suitable tissue and assay preparation; tumor-naïve approaches offer broader access but face background-signal discrimination. A negative result cannot establish cure. Earlier molecular detection creates an opportunity to test intervention, but lead time alone may lengthen the measured interval from detection to death without changing survival. Trials must determine whether ctDNA-directed escalation, de-escalation or earlier treatment improves outcomes without unnecessary toxicity. Standard adjuvant treatment or indicated surgery should not be withheld solely on an unvalidated biomarker result (21, 25).

## Artificial intelligence and model validation

AI and machine learning can combine molecular measurements with clinical and imaging variables, but complexity is not evidence of added value. A flexible model can learn specimen handling, recruitment site, age or disease-stage differences rather than a transportable cancer signal. Published PDAC liquid-biopsy models vary in cohort size, input features and validation methods (19, 38, 39). Comparisons should include a prespecified conventional multivariable model, such as penalized logistic regression, using the same data partitions and candidate information.

Patients, rather than individual aliquots or repeated samples, must be separated into training, tuning/validation and final test datasets. Feature selection, normalization, imputation, batch correction and hyperparameter optimization must be fitted within training folds; selecting biomarkers on the entire dataset before cross-validation leaks outcome information. Nested cross-validation can support tuning, but an untouched test set is still required for a final internal assessment. Multicenter recruitment followed by a random split does not constitute independent external validation.

Class imbalance should be handled within training data, with the test population retaining its intended-use distribution. Report sensitivity and specificity with confidence intervals, PPV/NPV at relevant prevalence, precision–recall performance when useful, and calibration plots, calibration intercept and slope or other suitable calibration measures. A model can discriminate well while systematically overestimating risk. Thresholds should reflect the consequences of missed cancers and false-positive procedures rather than be chosen solely to maximize the Youden index.

External evaluation should test a locked model in geographically or temporally distinct cohorts, with relevant benign controls and different laboratory batches. Interpretable feature summaries can identify reliance on implausible correlates, but feature importance is not a causal explanation and does not establish fairness or robustness. Reproducibility requires reporting the assay, preprocessing, missing-data handling, software versions, model specification and accessible code or a usable locked model where feasible. TRIPOD+AI provides a reporting framework (40).

A prospective “silent” evaluation can assess calibration and operational failure before results influence care. Subsequent clinical-impact studies should test a specified action triggered by the prediction and measure benefit and harm. PANLIPSY provides a protocol for prospective multimarker development and a separate validation phase (41); its planned results must not be presented as achieved performance. The review’s practical recommendation is to evaluate whether AI adds useful, reproducible information beyond standard clinical assessment, rather than assume that all multi-omic models need complex algorithms.

## Prospective evidence and clinical implementation

**Table 3** separates published prospective results from a development protocol. PANLIPSY plans multimarker discovery followed by blinded validation; METAPAC evaluates diagnostic discrimination in patients with pancreatic lesions; Groot and Bernard address longitudinal outcomes (25, 26, 37, 41). The older longitudinal studies are included as illustrative clinical-validation evidence, not as a claim to an exhaustive inventory of current trials. No current recruitment status is inferred from a publication alone.

PANLIPSY’s planned validation includes resectable PDAC and healthy controls, while benign pancreatic disease is represented in discovery (41). This design is valuable for independent classification but leaves performance against benign disease in the final validation setting unresolved. METAPAC provides a more clinically relevant diagnostic population, but its enrichment design still differs from screening. These distinctions explain why the word “prospective” alone does not determine clinical applicability.

Clinical utility should be evaluated against the care pathway that the assay is intended to modify. For diagnostic triage, relevant outcomes include time to diagnosis, cancers missed or delayed, repeat biopsies, unnecessary surgery, procedure complications and patient-reported burden. For surveillance, interval cancer rate and the proportion of clinically consequential resectable cancers are useful intermediate endpoints, but survival comparisons must address lead-time and length biases. For postoperative monitoring, the central question is whether biomarker-triggered treatment improves recurrence-free or overall survival and quality of life. None of the observational associations described here alone establishes that effect.

Decision-curve analysis can estimate net benefit over plausible action thresholds by weighting false positives against true positives; it should compare the new pathway with current practice, investigate-all and investigate-none strategies where relevant. It complements discrimination and calibration but is not a substitute for prospective clinical-impact evidence. Health-economic assessment should include assay and repeat-testing costs, failed samples, confirmatory imaging or biopsies, complications, treatment consequences, turnaround time and access. A modelled reduction in investigations is not observed cost-effectiveness. The studies highlighted here do not establish general cost-effectiveness of a PDAC liquid-biopsy pathway.

A pragmatic translational sequence is to specify the population and action, validate laboratory performance across sites, externally validate a locked assay or model, and test the resulting clinical pathway prospectively. Earlier-stage discovery studies should remain clearly labelled. The strongest implementation claim supported here concerns selected genotyping in established disease; diagnostic panels, high-risk surveillance additions and biomarker-directed postoperative interventions require further application-specific evidence (21, 22).

## Pre-analytical and analytical standardization

Collection and handling should be assay-specific and prospectively documented. Report tube type, blood volume, time and temperature before processing, centrifugation protocol, plasma or serum matrix, storage temperature and duration, and freeze–thaw cycles. Delayed processing can release leukocyte DNA; serum clotting and platelet activation can change DNA, RNA and EV profiles. Plasma and serum results should not be treated as interchangeable. Timing relative to biliary drainage, infection, surgery and systemic treatment should be recorded because these conditions can alter biological signals (21, 28, 41).

For ctDNA, report extraction method, input volume, DNA yield, unique molecular coverage rather than sequencing depth alone, error suppression, limit of blank, limit of detection and the decision threshold. Analytical detection limits must be distinguished from clinical sensitivity. Include controls for contamination, inter-run variation and clonal hematopoiesis where relevant. For CTCs, specify enrichment markers or physical selection criteria, recovery efficiency, the cellular definition used, counting reproducibility and reader blinding (15, 21, 28).

For EVs, isolation by ultracentrifugation, size-exclusion chromatography, precipitation or immunocapture can recover different fractions and co-isolate non-vesicular material. Report characterization and contaminant controls rather than assume equivalent “exosome” preparations (31). RNA assays require documentation of hemolysis assessment, extraction recovery, spike-ins, endogenous reference selection and normalization; a reference RNA must be stable in the intended population. Protein and metabolite assays require calibration, reagent-lot and matrix-effect assessment. Across platforms, randomize cases and controls across batches, use blinded replicates and external quality assessment, and establish inter-laboratory reproducibility before claiming a transferable cut-off (28, 41).

## Discussion

The evidence reviewed here supports a more differentiated assessment of liquid biopsy in PDAC than a single judgement about diagnostic accuracy. Its value depends on whether the clinical task is to detect an unsuspected cancer, resolve diagnostic uncertainty, identify an actionable alteration or monitor established disease. The central translational problem is that evidence supporting one of these tasks is often used to imply readiness for another. High discrimination between known cancers and selected controls establishes a rationale for further evaluation but does not establish that testing asymptomatic people or changing treatment will improve outcomes. Accordingly, the contribution of multimodal liquid biopsy should be judged against a defined clinical pathway and its existing alternatives (21, 22, 27, 28).

Apparent disagreement between biomarker studies is partly attributable to differences in the questions their designs can answer. A case-control study enriched for advanced PDAC may identify a strong cancer-associated signal while providing little information about detection of small, low-shedding lesions. Healthy controls also fail to reproduce the inflammatory, obstructive and metabolic conditions encountered during pancreatic investigations. Independent case-control validation, as demonstrated for the Mellby protein signature, is an advance beyond model development, but it does not remove this spectrum problem (35). Similarly, large recruitment in HELP-15 broadens the sampling base, whereas internal data splitting leaves uncertainty about performance in a new clinical and laboratory environment (34). The clinically relevant question is whether an assay retains useful discrimination at a prespecified threshold when applied to consecutive patients in its intended setting.

The findings therefore do not justify a hierarchy in which EVs or miRNAs are intrinsically superior to ctDNA or CTCs. Pooled estimates combine different targets, thresholds and patient populations, and the abundance of a circulating analyte is distinct from its tumor specificity (29, 32). A signal from host inflammation may improve classification in one cohort but reduce specificity against pancreatitis. Conversely, a mutation-based assay with limited sensitivity for early detection may remain informative for genotyping established disease. This distinction changes how conflicting results should be interpreted: an unsuccessful early-detection assay is not necessarily an uninformative monitoring assay, and a promising diagnostic signature is not automatically a predictive marker of treatment benefit. Biomarker selection should follow the clinical task rather than the highest published AUC.

Multimodal integration offers a plausible response to biological heterogeneity, but its incremental value must be demonstrated rather than assumed. Additional analytes can provide complementary information, yet they can also introduce correlated inflammatory signals, technical failures and additional fitted parameters. CA19–9 remains an important comparator because a new panel must improve decisions beyond information already available from routine assessment. METAPAC illustrates the need to examine the operating threshold: improved overall discrimination and specificity accompanied reduced sensitivity relative to CA19–9 (37). Whether such a trade-off is useful depends on the action triggered by the result. A strategy intended to avoid an invasive investigation needs particular scrutiny of false-negative consequences, while a test that triggers additional investigations must justify the burden of false positives. Neither an AUC increase nor statistical significance alone establishes an acceptable balance.

The distinction between screening and diagnostic evaluation is especially consequential. In an average-risk asymptomatic population, low disease prevalence can produce a large number of false positives despite apparently favorable test characteristics. High-risk surveillance offers a more focused evaluation setting, but inherited or familial risk does not make an unvalidated blood assay a replacement for MRI/MRCP or EUS (23, 24). Studies should determine whether adding the assay identifies clinically consequential disease earlier, reduces interval cancers or changes the burden of investigations. In patients who already have symptoms or indeterminate lesions, the priority is performance against relevant benign disease and integration with pathology and follow-up. These settings may provide clearer initial opportunities to test clinical benefit, but this is a translational judgement rather than evidence that a particular implementation strategy has already proved effective.

Longitudinal applications have a different evidential basis. Selected ctDNA genotyping can inform molecular assessment when tissue is insufficient or timely profiling is needed, whereas postoperative detection primarily provides information about recurrence risk (21, 25). These uses should not be grouped under a blanket statement that liquid biopsy is either established or entirely investigational. The discordant ctDNA and exosome-DNA findings reported by Bernard et al. further caution against assuming that all circulating measurements track treatment response equivalently (26). For postoperative surveillance, earlier molecular detection is clinically interesting only if it enables an effective intervention or a meaningful improvement in care. Without such evidence, earlier detection may change the timing of a diagnosis without changing the patient’s outcome. Association with recurrence is therefore insufficient to justify withholding standard therapy, abandoning indicated surgery or escalating treatment solely on the basis of an unvalidated assay.

AI can help combine these signals, but it can also amplify weaknesses in study design. When recruitment site, storage conditions or disease stage differ between cases and controls, a model may learn those differences instead of a reproducible cancer signature. Additional molecular features can increase this risk when the number of outcome events is limited. The most convincing evidence would therefore come from a locked assay and model evaluated independently across institutions, batches and clinically relevant subgroups, with calibration and decision consequences assessed alongside discrimination (39, 40). Comparisons with conventional regression are necessary to establish whether algorithmic complexity adds useful information. Reporting standards improve transparency, but compliance with a reporting checklist does not itself demonstrate transportability or clinical benefit.

Pre-analytical standardization is integral to this evaluation rather than a separate technical issue. Differences in processing delay, plasma or serum use, EV isolation and RNA normalization can alter which biological signal reaches the model. An apparently strong classifier may consequently be inseparable from the laboratory workflow in which it was developed. Standardization should encompass the specimen, assay, data-processing pipeline and positivity threshold, with reproducibility established across laboratories before a fixed clinical cut-off is transferred (28, 31, 41). This also has practical consequences: a panel that requires several specialized platforms may offer less usable benefit than a simpler assay if turnaround time, sample failure or access prevents its result from influencing the intended decision. These operational considerations should be measured alongside analytical performance.

Prospective studies represent progress, but their design must match the implementation claim. PANLIPSY provides an organized framework for multimarker development and independent classification; its planned final validation against healthy controls does not resolve every question about differentiation from benign pancreatic disease (41). METAPAC addresses a more clinically relevant lesion-evaluation population, although an enriched diagnostic study cannot establish population screening benefit (37). Together, these examples support a sequence of application-specific analytical validation, independent clinical validation and prospective assessment of a predefined management strategy. Decision-curve and economic analyses can help identify plausible benefit and resource implications, but trials of clinical impact are needed to establish whether the pathway reduces missed cancers, unnecessary procedures or treatment-related harm. Cost-effectiveness should include downstream investigations and treatment consequences, not merely the price of the blood test.

This review also has limitations that constrain the strength of its synthesis. The examples discussed cannot establish the comparative effectiveness of platforms, and the purposive selection and heterogeneity of the included evidence limit comparative inference. These limitations favor cautious, application-specific conclusions rather than broad recommendations for adoption. The principal research priority emerging from this synthesis is to test a reproducible assay within a clearly specified clinical decision: who is tested, when the sample is taken, what threshold triggers action and whether that action improves outcomes. Such evidence would allow liquid biopsy to progress from promising classification performance towards a justified role in PDAC care.

## Conclusion

Liquid biopsy in PDAC should be judged by its intended population, clinical decision and consequences. Selected plasma genotyping can complement tissue-based molecular profiling, while diagnostic panels and longitudinal biomarkers provide promising but uneven evidence of clinical validity. Published AUCs do not support ranking entire biomarker classes, and prognostic associations do not establish benefit from biomarker-directed treatment. The priorities are assay reproducibility, independent validation against relevant comparators, calibrated models and prospective evaluation of patient outcomes, harms and costs. These steps are necessary before promising blood-based signals can become reliable components of PDAC care.

## References

[1] Yu W Zhou D Meng F Wang J Wang B Qiang J . The global, regional burden of pancreatic cancer and its attributable risk factors from 1990 to 2021. BMC Cancer. (2025) 25:186. doi: 10.1186/s12885-025-13471-y 39891086 PMC11786447

[2] Liu C Liu P Liu X Niu W Wu P Yu J . Global, regional, and national burden and trends of pancreatic cancer, 1990-2021: a systematic analysis for the Global Burden of Disease Study 2021. Front Oncol. (2025) 15:1671856. doi: 10.3389/fonc.2025.1671856 41256325 PMC12620227

[3] Leiphrakpam PD Chowdhury S Zhang M Bajaj V Dhir M Are C . Trends in the global incidence of pancreatic cancer and a brief review of its histologic and molecular subtypes. J Gastrointest Cancer. (2025) 56:71. doi: 10.1007/s12029-025-01183-2 39992560

[4] Zottl J Sebesta CG Tomosel E Sebesta MC Sebesta C . Unraveling the burden of pancreatic cancer in the 21st century: Trends in incidence, mortality, survival, and key contributing factors. Cancers (Basel). (2025) 17:1607. doi: 10.3390/cancers17101607 40427106 PMC12110279

[5] Zhang L Sanagapalli S Stoita A . Challenges in diagnosis of pancreatic cancer. World J Gastroenterol. (2018) 24:2047–60. doi: 10.3748/wjg.v24.i19.2047 29785074 PMC5960811

[6] Shen MJ Jamali A Katona BW . Challenges of early detection of pancreatic cancer. J Clin Invest. (2025) 135:e191942. doi: 10.1172/JCI191942 41090355 PMC12520692

[7] Robatel S Schenk M . Current limitations and novel perspectives in pancreatic cancer treatment. Cancers (Basel). (2022) 14:985. doi: 10.3390/cancers14040985 35205732 PMC8870068

[8] Carrara S Bevere M Delle Cave D Gentiluomo M Esposito I Luchini C . Liquid biopsy in pancreatic cancer: current advances, limitations, and future perspectives. Dig Liver Dis. (2026) 58:726–36. doi: 10.1016/j.dld.2026.03.002 41866258

[9] Ramírez-Maldonado E López Gordo S Major Branco RP Pavel MC Estalella L Llàcer-Millán E . Clinical application of liquid biopsy in pancreatic cancer: A narrative review. Int J Mol Sci. (2024) 25:1640. doi: 10.3390/ijms25031640 38338919 PMC10855073

[10] Kamyabi N Bernard V Maitra A . Liquid biopsies in pancreatic cancer. Expert Rev Anticancer Ther. (2019) 19:869–78. doi: 10.1080/14737140.2019.1670063 31533487 PMC6824837

[11] Amaral MJ Oliveira RC Donato P Tralhão JG . Pancreatic cancer biomarkers: Oncogenic mutations, tissue and liquid biopsies, and radiomics-a review. Dig Dis Sci. (2023) 68:2811–23. doi: 10.1007/s10620-023-07904-6 36988759 PMC10293428

[12] Bararia A Chakraborty P Roy P Chattopadhay BK Das A Chatterjee A . Emerging role of non-invasive and liquid biopsy biomarkers in pancreatic cancer. World J Gastroenterol. (2023) 29:2241–60. doi: 10.3748/wjg.v29.i15.2241 37124888 PMC10134423

[13] Toledano-Fonseca M Brozos-Vázquez E García-Ortiz MV Costa-Fraga N Díaz-Lagares Á Rodríguez-Ariza A . Unveilling the potential of liquid biopsy in pancreatic cancer for molecular diagnosis and treatment guidance. Crit Rev Oncol Hematol. (2025) 212:104807. doi: 10.1016/j.critrevonc.2025.104807 40517975

[14] Qi ZH Xu HX Zhang SR Xu JZ Li S Gao HL . The significance of liquid biopsy in pancreatic cancer. J Cancer. (2018) 9:3417–26. doi: 10.7150/jca.24591 30271504 PMC6160675

[15] Stosic K Senar OA Tarfouss J Bouchart C Navez J Van Laethem JL . A comprehensive review of the potential role of liquid biopsy as a diagnostic, prognostic, and predictive biomarker in pancreatic ductal adenocarcinoma. Cells. (2023) 13:3. doi: 10.3390/cells13010003 38201207 PMC10778087

[16] Wang K Wang X Pan Q Zhao B . Liquid biopsy techniques and pancreatic cancer: diagnosis, monitoring, and evaluation. Mol Cancer. (2023) 22:167. doi: 10.1186/s12943-023-01870-3 37803304 PMC10557192

[17] Zhao Y Tang J Jiang K Liu SY Aicher A Heeschen C . Liquid biopsy in pancreatic cancer - current perspective and future outlook. Biochim Biophys Acta Rev Cancer. (2023) 1878:188868. doi: 10.1016/j.bbcan.2023.188868 36842769

[18] Chen X Hu X Liu T . Development of liquid biopsy in detection and screening of pancreatic cancer. Front Oncol. (2024) 14:1415260. doi: 10.3389/fonc.2024.1415260 38887233 PMC11180763

[19] Hou J Li X Xie KP . Coupled liquid biopsy and bioinformatics for pancreatic cancer early detection and precision prognostication. Mol Cancer. (2021) 20:34. doi: 10.1186/s12943-021-01309-7 33593396 PMC7888169

[20] Dubrovsky G Ross A Jalali P Lotze M . Liquid biopsy in pancreatic ductal adenocarcinoma: A review of methods and applications. Int J Mol Sci. (2024) 25:11013. doi: 10.3390/ijms252011013 39456796 PMC11507494

[21] Pascual J Attard G Bidard FC Curigliano G De Mattos-Arruda L Diehn M . ESMO recommendations on the use of circulating tumour DNA assays for patients with cancer: a report from the ESMO Precision Medicine Working Group. Ann Oncol. (2022) 33(8):750–68. doi: 10.1016/j.annonc.2022.05.520 35809752

[22] Conroy T Pfeiffer P Vilgrain V Lamarca A Seufferlein T O'Reilly EM . Pancreatic cancer: ESMO clinical practice guideline for diagnosis, treatment and follow-up. Ann Oncol. (2023) 34:987–1002. doi: 10.1016/j.annonc.2023.08.009 37678671

[23] Takahashi K Ono Y Taniue K Patra KC Yamamoto T Fujiya M . Liquid biopsy for early pancreatic cancer detection: Why has it not yet worked? Cancers (Basel). (2026) 18:525. doi: 10.3390/cancers18030525 41681997 PMC12896831

[24] Goggins M Overbeek KA Brand R Syngal S Del Chiaro M Bartsch DK ; International Cancer of the Pancreas Screening (CAPS) consortium. Management of patients with increased risk for familial pancreatic cancer: updated recommendations from the International Cancer of the Pancreas Screening (CAPS) Consortium. Gut. (2020) 69(1):7–17. doi: 10.1136/gutjnl-2019-319352 31672839 PMC7295005

[25] Groot VP Mosier S Javed AA Teinor JA Gemenetzis G Ding D . Circulating tumor DNA as a clinical test in resected pancreatic cancer. Clin Cancer Res. (2019) 25:4973–84. doi: 10.1158/1078-0432.CCR-19-0197 31142500 PMC7403524

[26] Bernard V Kim DU San Lucas FA Castillo J Allenson K Mulu FC . Circulating nucleic acids are associated with outcomes of patients with pancreatic cancer. Gastroenterology. (2019) 156:108–118.e4. doi: 10.1053/j.gastro.2018.09.022 30240661 PMC6434712

[27] Reese KL Pantel K Smit DJ . Multibiomarker panels in liquid biopsy for early detection of pancreatic cancer - a comprehensive review. J Exp Clin Cancer Res. (2024) 43:250. doi: 10.1186/s13046-024-03166-w 39218911 PMC11367781

[28] Munnings R Gibbs P Lee B . Evolution of liquid biopsies for detecting pancreatic cancer. Cancers (Basel). (2024) 16:3335. doi: 10.3390/cancers16193335 39409954 PMC11475855

[29] Zheng Z Lu Z Yan F Song Y . The role of novel biomarkers in the early diagnosis of pancreatic cancer: A systematic review and meta-analysis. PloS One. (2025) 20:e0322720. doi: 10.1371/journal.pone.0322720 40408437 PMC12101772

[30] Balaraman AK Moglad E Afzal M Babu MA Goyal K Roopashree R . Liquid biopsies and exosomal ncRNA: Transforming pancreatic cancer diagnostics and therapeutics. Clin Chim Acta. (2025) 567:120105. doi: 10.1016/j.cca.2024.120105 39706249

[31] Welsh JA Goberdhan DCI O’Driscoll L Buzas EI Blenkiron C Bussolati B . Minimal information for studies of extracellular vesicles (MISEV2023): From basic to advanced approaches. J Extracell Vesicles. (2024) 13:e12404. doi: 10.1002/jev2.12404 38326288 PMC10850029

[32] Zhu Y Zhang H Chen N Hao J Jin H Ma X . Diagnostic value of various liquid biopsy methods for pancreatic cancer: A systematic review and meta-analysis. Med (Baltimore). (2020) 99:e18581. doi: 10.1097/MD.0000000000018581 32011436 PMC7220382

[33] Malakian A Modestino A Bueno J Machireddy A Ware J Hamilton S . Liquid biopsy differentiation of pancreatic cancer from non-cancerous pancreatic disease using dielectrophoresis-recovered nanoparticles carrying cell-free DNA and protein biomarkers. Small (Weinheim der Bergstrasse). (2026) 22:e02532. doi: 10.1002/smll.202502532 41947711 PMC13206421

[34] Lee JC Kang SW Sim EJ Bae JS Koo SM Byoun MS . Novel mRNA biomarker-based liquid biopsy for the detection of resectable pancreatic cancer. BMC Cancer. (2025) 25:762. doi: 10.1186/s12885-025-14124-w 40269781 PMC12016232

[35] Mellby LD Nyberg AP Johansen JS Wingren C Nordestgaard BG Bojesen SE . Serum biomarker signature-based liquid biopsy for diagnosis of early-stage pancreatic cancer. J Clin Oncol. (2018) 36:2887–94. doi: 10.1200/JCO.2017.77.6658 30106639 PMC6161836

[36] Wang X Che Y Liu S Ma B Liu Y Lei J . Single-molecule liquid biopsy detects low- and high-abundance protein markers simultaneously for pancreatic cancer diagnosis. Anal Chem. (2025) 97:8385–93. doi: 10.1021/acs.analchem.4c07031 40194997

[37] Mahajan UM Oehrle B Goni E Strobel O Kaiser J Grützmann R . Validation of two plasma multimetabolite signatures for patients at risk of or with suspected pancreatic ductal adenocarcinoma (METAPAC): a prospective, multicentre, investigator-masked, enrichment design, phase 4 diagnostic study. Lancet Gastroenterol Hepatol. (2025) 10:634–47. doi: 10.1016/S2468-1253(25)00056-1 40388948

[38] Nirob SR Morol MK Rahman D Hui LT Nandi D Rahman M . Early multi-cancer detection using liquid biopsy: emerging biomarkers and clinical strategies. Front Oncol. (2026) 16:1877911. doi: 10.3389/fonc.2026.1877911 42490792 PMC13375447

[39] Ku J Singhal M Burnette M Hegazy SA . Machine learning and artificial intelligence in liquid biopsy-based early detection of pancreatic cancer: a scoping review. BJC Rep. (2026) 4(1):26. doi: 10.1038/s44276-026-00232-y 42168331 PMC13195041

[40] Collins GS Moons KGM Dhiman P Riley RD Beam AL Van Calster B . TRIPOD+AI statement: updated guidance for reporting clinical prediction models that use regression or machine learning methods. BMJ. (2024) 385:e078378. doi: 10.1136/bmj-2023-078378 38626948 PMC11019967

[41] Bardol T Dujon AM Taly V Dunyach-Remy C Lavigne JP Costa-Silva B . Early detection of pancreatic cancer by liquid biopsy "PANLIPSY": a French nation-wide study project. BMC Cancer. (2024) 24:709. doi: 10.1186/s12885-024-12463-8 38853244 PMC11163786

